# The saccade main sequence revised: A fast and repeatable tool for oculomotor analysis

**DOI:** 10.3758/s13428-020-01388-2

**Published:** 2020-07-08

**Authors:** Agostino Gibaldi, Silvio P. Sabatini

**Affiliations:** 1 School of Optometry and Vision Science, University of California at Berkeley, 380 Minor Lane, CA USA; 2 Department of Informatics, Bioengineering, Robotics and System Engineering, University of Genoa, Via All’Opera Pia, 13, Genoa, 16145 Italy

**Keywords:** Saccade main sequence, Eye movements, Binocular eye tracking, Saccade trajectory

## Abstract

Saccades are rapid ballistic eye movements that humans make to direct the fovea to an object of interest. Their kinematics is well defined, showing regular relationships between amplitude, duration, and velocity: the saccadic ’main sequence’. Deviations of eye movements from the main sequence can be used as markers of specific neurological disorders. Despite its significance, there is no general methodological consensus for reliable and repeatable measurements of the main sequence. In this work, we propose a novel approach for standard indicators of oculomotor performance. The obtained measurements are characterized by high repeatability, allowing for fine assessments of inter- and intra-subject variability, and inter-ocular differences. The designed experimental procedure is natural and non-fatiguing, thus it is well suited for fragile or non-collaborative subjects like neurological patients and infants. The method has been released as a software toolbox for public use. This framework lays the foundation for a normative dataset of healthy oculomotor performance for the assessment of oculomotor dysfunctions.

## Introduction

When scanning the surrounding environment, human eyes make two to three fixations per second and move very quickly between each fixation with a saccadic eye movement. Since the very beginning of eye movement research, the kinematics of eye movements has been investigated with a variety of measurement techniques: suction contact lenses (Yarbus, 1967), scleral search coils (Robinson, 1964; Fuchs, 1967), electro-oculography (Becker & Fuchs, 1969; Baloh et al., 1975), limbus tracking (Stark et al., 1962; Bahill et al., 1975). All researchers agreed that eye-movement patterns are highly stereotyped: the duration and peak velocity of the saccades increase as the magnitude of the saccades increases (Yarbus, 1967; Robinson, 1964; Fuchs, 1967; Bahill et al., 1975). Particularly, the increase is linear for short saccades while the speed asymptotically approaches a saturated value for large saccades. Borrowing a term from astronomy, Bahill and colleagues defined this relation as the *main sequence* for saccadic eye movements (Bahill et al., 1975).

The main sequence has been proven a simple and powerful tool to investigate eye movements (see Leigh & Kennard, 2004; Ramat et al.,, (2006) as review). In basic research, it has been used to: 1) design and test neural models of saccadic eye movement control (Bahill et al., 1975; Becker, 1989; Robinson et al., 1993; Fuchs et al., 1993; Quaia et al., 1999; Jagadisan & Gandhi, 2017), 2) evaluate the effect of blinks (Khazali et al., 2016; Jagadisan & Gandhi, 2017), 3) compare different experimental paradigms (Smit et al., 1987), 4) study eye movement adaptation (Optican & Robinson, 1980), 5) characterize micro-saccades (Martinez-Conde, 2004, 2009), 6) infer the dynamics of perceptual decision-making (Seideman et al., 2018), and 7) even evaluate the effect of opioids (Grace et al., 2010). In clinical research, the main sequence has been used as diagnostic tool to assess the integrity of the saccadic system. In fact, certain deviations from normal performance can be used as markers to a specific disease (Troost & Daroff, 1977; Frohman et al., 2002; Leigh & Kennard, 2004; Ramat et al., 2006). The main sequence has been successfully used to investigate eye movements dysfunctions in patients with 1) palsy of extra-ocular muscles (Metz et al., 1970; Garbutt et al., 2003), 2) myasthenia gravis (Yee et al., 1976), 3) cerebellar disorder (Selhorst et al., 1976), 4) ocular progressive supra-nuclear palsy (Troost & Daroff, 1977), 5) multiple sclerosis (Frohman et al., 2002; Bijvank et al., 2019), 6) spino-cerebellar and cerebellar ataxia (Federighi et al., 2011, 2017, and 7) Parkinson’s disease (Otero-Millan et al., 2013).

Despite the effectiveness of the approach, there are several issues to be taken into account for reliable and repeatable measurements of eye performance. First, kinematic parameters are usually obtained using numerical and differential method, which are implicitly sensitive to sampling frequency and noise in the measurement. The original methods required sampling frequency greater than 330 Hz (Bahill et al.,, 1982, 1983; Juhola et al.,, 1985; Leigh & Zee, 2015), although 1000 Hz was considered desirable (Bahill et al., 1975, 1982, 1983) to obtain reliable numerical measurements. This is done to prevent underestimating peak velocity and duration, and misjudging the main sequence. On the other hand, subsequent and more robust algorithms have been shown to be effective down to 200 Hz of sampling frequency (Inchingolo & Spanio, 1985; Federighi et al., 2011). Second, there is no general consensus about the mathematical model describing the main sequence. A power law rule was first used to describe the non-linear growth of peak velocity with saccade eccentricity (Yarbus, 1967; Lebedev et al., 1996). In fact, peak velocity reaches a saturated value for large saccades, similarly to an exponential function (Bahill et al., 1975; Baloh et al., 1975; Smit et al., 1987; Leigh & Zee, 2015).

Aiming at an actual and effective use of the main sequence in clinics, one should consider models characterized by robustness and generalization capability, in order to provide a tool to compare inter- and intra-subject variability.

Third, recent years have seen a rapid advancement and widespread use of eye-tracking technology (see Gibaldi et al., (2017b) for review). There are a number of eye-tracking devices, available off the shelf, that generally work at sampling frequencies much lower than 330 Hz. These devices are not meant simply for consumer applications, like gaming, virtual reality, and human–computer interaction (e.g., Tobii 4C, TheEyeTribe (discontinued)), but can be also dedicated to research (e.g., Pupil Labs, Tobii Pro devices like Glassess2, Nano and XL, the GazePoint GP3).

In this work, we provide a standard methodology to characterize oculomotor performance. Eye movements are measured either with sequential lab stimulation or during a quick and non-fatiguing procedure of natural image exploration. The parameters of saccade kinematics are obtained using a modeling approach, so that their estimates are robust to noise and are not affected by low sampling frequencies down to $\sim $50 Hz. The main sequence is then characterized using different models proposed in the literature. Specifically, we focus on a one-parameter model to obtain a simple and compact representation of oculomotor performance to allow for a direct numerical comparison with normative data. The proposed methodology is robust and repeatable but also non-fatiguing, fast, and easy to use. The approach has been released in the form of a software toolbox available for public use (see Appendix B).

## Materials and methods

The proposed methodology is based on two steps of analysis. We will first describe methods to obtain robust estimations of saccadic kinematic parameters, reliable also for low temporal sampling frequencies. Then, we will evaluate the estimation capability of the selected models with an extensive battery of tests, so as to provide contextual guidelines for model selection. The methodology will be tested on two datasets, one containing eye traces acquired during a sequential and controlled lab stimulation (Exp. 2), and a second one acquired during free visual exploration of a natural images (Exp. 2).

### Evaluating saccade parameters

A number of parameters can be directly extracted from a single saccade to analyze the oculomotor performance (Becker, 1989). Here we will focus on *start* and *end points*, *duration*, *amplitude* and *peak velocity*.

A careful, repeatable, and robust estimation of these parameters is required for their use, either in clinics or in research.

#### Start and End Point

**Fig. 1:**
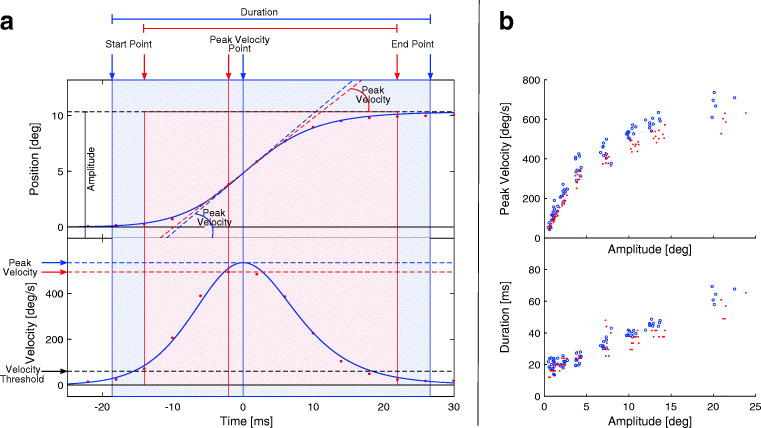
**A. Example of model fitting and parameter computation for a single saccade.** Position (top) and velocity (bottom) profiles of a saccade. The red dots represent samples from the eye-tracking device (Eyelink II). A velocity threshold of 50 deg/s is used to compute the start and end points of the saccade, from which the amplitude and duration are determined. The velocity profile is computed using a two-point central difference algorithm, and the peak velocity is the maximum sample in the profile. The blue solid line is computed using the fitting approach. The start and end point are the 2% and 98% of the amplitude, from which the amplitude and duration. The velocity profile is an analytic derivative of the fitted Sigmoid, and provides sub-sample resolution to compute peak velocity. **B. Example of the main sequence for amplitude-peak velocity (top) and for amplitude-duration (bottom).** Red dots represent the kinematic parameters computed with a standard sampling approach, while blue dots are the same data but computed with the fitting approach

A standard methodology consists in using a velocity/acceleration threshold to mark the start and end points of the saccade. Common values for the velocity threshold can range between 5 and 50 deg/s (Baloh et al., 1975; Inchingolo & Spanio, 1985; Federighi et al., 2011), using lower values for oculomotor studies and higher values for cognitive studies. This methodology results in a systematic underestimation of the saccadic duration (see Fig. 1). If reducing the threshold might seem a reasonable way to increase accuracy, a low threshold would be more sensitive to noise in the data (Bahill et al., 1981). Moreover, in case of a saccadic over/undershoot, this method would not be able to distinguish between the main and the corrective saccade, resulting in a delayed end point and overestimating saccade duration (Bahill et al., 1975). The problem would be mitigated using a *relative* threshold, like the 5% of the saccadic peak velocity (Bahill et al., 1981).

Nevertheless, any threshold approach would be affected by sampling frequency. The threshold crossing would very seldom match the time of a sample acquisition, but would happen between samples, resulting in an over/underestimation of saccade duration (Andersson et al., 2010).

#### Duration

The measurement of the saccadic duration will rely on the estimated start and end points of the saccade. Accordingly, it is equally affected by the same problems of thresholding and sampling frequency. Exemplifying, on a saccade of 10 degrees, a sampling frequency of 200 Hz would result in an error ± 5 ms, which is roughly 12% of its duration. Besides, if we reduce the sampling frequency to 50 Hz, the error would become ± 20 ms which is close to 50% of the saccade duration.

#### Amplitude

Similarly, the estimation of saccadic amplitude will be derived from the estimated start and end points of the saccade. The threshold approach will result in a systematic underestimation of the amplitude (see Fig. 1). Nevertheless, saccadic kinematics is characterized by a smooth acceleration and deceleration, so the movement performed near the start and end point of the saccade can be considered negligible with respect to the whole amplitude.

#### Peak Velocity

The easiest measure of eye velocity is a differentiation of the eye position signal using a two-point central difference algorithm (Schmidt et al., 1979):

1 $$ V_{p}(t) = \frac{\vec{X}(t) - \vec{X}(t-1)}{\Delta t} $$

This algorithm has shown to provide reliable results only under the condition of a sampling frequency of at least 330 Hz (Bahill et al.,, 1982, 1983; Juhola et al.,, 1985; Leigh & Zee, 2015), even if 1000 Hz was considered desirable (Bahill et al., 1975, 1982, 1983). This technique has two main drawbacks. First, the numerical derivative is intrinsically highly sensitive to noise. Depending on the device, it might be difficult or even impossible to characterize and remove the measurement noise. Furthermore, filtering the noise will affect the estimated velocity, and specifically the peak velocity. Second, the method is highly sensitive to sampling frequency. The instant of peak velocity usually falls between two samples, resulting in an underestimation of the peak magnitude (see Fig. 1).

Over the years, more complex and robust techniques have been proposed, like an eight-point central difference derivative algorithm (Inchingolo & Spanio, 1985; Federighi et al., 2011). These techniques have been proven enough robust to allow downsampling to 200 Hz (Inchingolo & Spanio, 1985; Juhola et al., 1985). A more recent work (Wierts et al., 2008) showed how a low sampling frequency can be effective for large saccades.

### Saccade fitting

Starting from the seminal studies in the field (Robinson, 1964; Bahill et al., 1975; Baloh et al., 1975), saccadic movements have been considered to be highly stereotyped. The eye starts moving smoothly, has an intense acceleration and a less intense deceleration to the end point of the trajectory.

From this perspective, it is reasonable to exploit model fitting to describe the saccadic trajectory. A possible approach is to fit the velocity profile (e.g., see Smit et al., 1987), but it is worth considering that velocity is a derived measurement that amplifies all the possible sources of noise. Here, we propose to directly fit a model to the spatial trajectory of the eyes. Such an approach is expected to be implicitly less prone to measurement noise, since the fitting algorithm makes it more robust to outlier samples. The saccade profile is fitted using a Sigmoid function in the form of the Hill’s Equation (Goutelle et al., 2008):

2 $$ \hat{y}(t)=E_{0} + (E_{\textsc{max}}-E_{0})\frac{t^{\alpha}}{(E_{50}+t)^{\alpha}}  $$

The trend of the Sigmoid curve is well suited to describe many natural processes that move from a steady state, accelerating rapidly and decelerating smoothly while approaching a saturated value. In our formulation *E*_0_ and *E*_max_ are the start and end point of the saccade, *E*_50_ is the time of half saturation, e.g., half of saccade trajectory, and *α* is a nonlinear parameter defining the slope of the curve. The fitting is performed using a Levemberg–Marquadt nonlinear least squares minimization algorithm. The reliability of the fitting is enhanced by providing a plausible initial estimate of Sigmoid parameters (Gibaldi et al., 2015).

This model provides a compact representation of the saccade profile, and it has been proven functional for an accurate identification of the start and end point of the saccade and to measure intra-saccadic vergence (Gibaldi & Banks, 2019), as well as to provide fine kinematic characterization of eye dominance (Gibaldi et al., 2016). The proposed model fitting approach presents a number of advantages: 1) saccade kinematics are obtained by analytical solutions rather than by numerical methods, which are prone to measurement noise; 2) the procedure is implicitly robust to outlier samples; 3) analytical solutions are relatively independent of sampling frequency and are able to provide sub-sample resolution.

### Models of the main sequence

Different models have been proposed to describe the main sequence, reported in Table 1, but no general consensus has been reached towards one model or another. In this work, we specifically focus on the relation between amplitude and peak velocity.

**Table 1:** Models used to fit the main sequence

1-Parameter	2-Parameter	3-Parameter
slope	line	cubic
*y* = *ax*	*y* = *ax* + *b*	*y* = *ax*^2^ + *bx* + *c*
sqrt (Lebedev et al., 1996)	power law (Yarbus, 1967; Lebedev et al., 1996; Garbutt et al., 2003)	exponential (Bahill et al., 1975; Baloh et al., 1975; Leigh & Zee, 2015)
$y = V \sqrt {x}$	*y* = *mx*^*V*^	$y = V(1-e^{-\frac {(x - A0)}{k}})$
fixed sqrt	log-log	sigmoid
$y = VA + V\sqrt {x - A_{th}}$	*y* = *e*^*Vlog*(*x*)+*Q*^	$y = \frac {A_{max}}{(1+(V50/x)^{\alpha })}$
*V* *A* = *V*_*peak*_(*A*_*th*_)		

The Table reports the equations of the nine models we assessed as estimator of the saccadic main sequence. These models have been grouped in three categories, i.e., models using one, two, or three parameters

A power law model (power law) is quite effective in describing the non-linear growth of peak velocity with saccade amplitude (Yarbus, 1967; Lebedev et al., 1996), and it has been successfully used to identify reduced performance on patients (Garbutt et al., 2003). Peak velocity increases with saccadic amplitude, and reaches a saturated value for saccades larger than 15 to 20 degrees. This trend can be well described by an exponential function (exponential). This model has been extensively used either in research (Bahill et al., 1975; Baloh et al., 1975; Smit et al., 1987; Leigh & Zee, 2015) and in clinics (Ramat et al., 2006; Federighi et al., 2011; Federighi et al., 2017). The trend is well modelled also by a Sigmoid equation (Goutelle et al., 2008) (sigmoid), even if it is generally not used in eye movement research. Another model, extensively used in literature, requires a double logarithmic transformation (log-log) (Bahill et al.,, 1975, 1981, Bollen et al.,, 1993; Garbutt et al.,, 2003). In this space, the main sequence becomes linear in a range of approximately 1–15 degrees. A relatively newer approach uses a simpler model (Lebedev et al., 1996), a square root equation (sqrt), to increase the robustness of the estimated main sequence. An extension of this model (fixed sqrt) exploits constants computed directly from the data, like the average peak velocity for 1-degree saccades. Furthermore, for small saccades the relationship between amplitude and peak velocity is roughly linear. We then used a simple slope (slope), but also a line equation (line) to fit the data. Finally, we also tested a cubic equation (cubic) to assess the usability of simple polynomial functions.

While comparing these models, one should be aware that a single data set might provide a limited perspective of model performances. An effective model should not just be robust and reliable, but should also allow experimenters to assess inter- and intra-subject variability, or to compare different experimental conditions. To this aim, we grouped the selected models with respect to the numbers of parameters, from one to three, as a measure of their complexity (see Table 1). Aiming at using compact models, we excluded those with a larger number of parameters (e.g., see Duchowski et al., 2017).

### Experimental design

#### Subjects

Nine subjects (six females and three males), ages 24–39 years (average 29.5, SD 5.1), participated. All but one were unaware of the experimental hypotheses. The subject protocol was approved by the Institutional Review Board at the University of California, Berkeley. All subjects gave informed consent before starting the experiment.

#### Experimental setup

Subjects sat in front of a large frontoparallel LCD screen (125 × 77cm) with HD resolution (1920 × 1080 pixels). A bite bar was used to stabilize the subject’s head. A custom sighting device was used to accurately position the eyes relative to the screen (Hillis & Banks, 2001), so that the midpoint of the subject’s inter-ocular axis was aligned with the center of the screen. The distance from the display screen was 100 cm. The experiment was performed in a dark room, with the screen being the only light source. The binocular gaze direction was measured with a head-mounted eye tracker (Eyelink II), using pupil and corneal reflections at 250 Hz. The visual stimuli were presented using Matlab with the Psychtoolbox (Brainard, 1997; Kleiner et al., 2007) and a toolbox for integration of the Eyelink (Cornelissen et al., 2002).

#### Eye movement calibration

A 13-point calibration procedure was first used to calibrate the eye tracker at the beginning of each session. The calibration targets subtended 0.6 degrees. The calibration area was adapted depending on the portion of the screen actually used in the experiment (Gibaldi et al., 2017b; Canessa et al., 2012). The calibration was followed by a nine-point validation procedure. Calibration was repeated to obtain a mean error less than 0.5 degrees, to ensure accuracy. The calibration target was designed to match the luminance of the experiment target, in order to improve eye-tracking accuracy (Drewes et al., 2012). After calibration, subject initiated stimulus presentation by button press.

#### Experiment 1: Sequential saccade testing

Visual stimulation was provided as a Maltese cross at different location on the screen. The cross covered 1 degree of visual field. At each trial, the target was first shown in front of the dominant eye. After 0.6 − 1 sec, the cross jumped left or right to a peripheral position and remained there for 1sec. The subject was instructed to follow the target as quickly and accurately as possible. The tested eccentricities were ± 1, ± 2, ± 4, ± 8, ± 12, ± 16 or ± 24degrees. The peripheral target was presented ten times at each eccentricity, for a total of 140 trials per session. To discourage anticipatory movements, the presentation order was random and the time interval between a button press and the displacement of the target was variable.

In order to evaluate the test-retest reliability, each subject repeated the experiment on two different days. The test was performed approximately at the same hour (between 9:00am and 10:00am), to reduce possible differences in the oculomotor performance due to fatigue (Schmidt et al., 1979; Galley, 1989; Bollen et al., 1993; Straube et al., 1997; Di Stasi et al., 2013).

#### Experiment 2: Free gaze exploration

Visual stimulation was provided using rendered images of natural environments. Similarly to Exp. 2, the session started with a calibration procedure for the eye tracker. The calibration encompassed the whole screen area. After that, subjects initiated stimulus presentations with a button press. At each trial, a Maltese cross was first shown in the center of the screen for 1 s. The visual stimuli consisted in twenty naturalistic scenes of peripersonal space (Gibaldi et al., 2017a; Canessa et al., 2017). Each image was presented for 20 s on the screen, while recording eye movements with the eye tracker. The subject was instructed to explore the scene with the gaze.

### Data analysis

#### Saccade detection

The gaze data obtained by the two experiments were analyzed in the following way. The pixel position on screen was first transformed in azimuth and elevation angles. A preliminary estimation of eye velocity and acceleration was performed using a two-points central difference algorithm. Saccades were detected considering a threshold of 20 deg/s on the velocity traces. Next, we marked the preceding and the following fixations. The trajectory of the saccade was then computed as the straight motion from one fixation to the next. For Exp. 2 we considered only the first saccade after target onset, disregarding possible corrective saccades and mis-fixations. For Exp. 2, we defined a threshold of 1 degree of eccentricity to detect possible micro-saccades (Martinez-Conde et al., 2009), and we discarded them from the dataset. The saccade kinematic parameters were then computed using either absolute thresholds or using the proposed Sigmoid fitting.

#### Varying the sampling frequency

The original gaze data we collected with a sampling frequency of 250 Hz. In order to be able to make a comparison between the proposed processing procedures on data sampled at lower frequencies, the gaze data were then sub-sampled at increasing factors from 1:2 to 1:8. The resulting data have a sampling frequency of 125, 83.3, 62.5, 50, 41.7, 35.7, and 31.3 Hz.

Accordingly, we assessed the robustness of the computed saccadic parameters at decreasing sampling frequencies, specifically for *amplitude*, *duration* and *peak velocity* of the saccade. We used gaze data from Exp. 2, assuming the parameters computed at 250 Hz as the gold standard. We then used a two-sided Wilcoxon rank-sum test to compare the medians of the parameters measured on the original and sub-sampled traces.

#### Model fitting approach

The fitting of a parametric model to the main sequence is usually performed on a large set of saccades. This ensures that the fitted model does not depend on possible outliers present in the data, and that the fitting is actually representative of the subject’s performance. Also, depending on the experimental procedure, saccadic range might vary considerably. While in natural viewing most of saccades are shorter than 15 degrees (Sprague et al., 2015), lab tests for main sequence usually require saccades of larger amplitudes, even up to 90 degrees (Baloh et al., 1975). It is worth considering that, depending on the pathology, patients might not be able to stand long and fatiguing procedures. Similarly, patients may have difficulties in initiating saccades and the movements themselves may be small, preventing the possibility to perform comparisons with data sets from control subjects (Garbutt et al., 2003).

We believe it is necessary to evaluate the reliability of model fitting to perform a robust and reliable estimation of the main sequence. To this purpose, we used a bootstrap analysis to obtain statistics of the goodness-of-fit (adjusted coefficient of variation, *R*^2^), and of the repeatability of the measurement (Minimum Absolute Percentage Error, MAPE Tofallis 2015). Each boot size was repeated 1000 times to obtain statistics of the estimator performance. The MAPE was computed between each of the fitted curve and every other curve in the boot. It represents the percentage change between two estimations performed on two different bootstrap sample. Note that a high value of *R*^2^ generally represents a good fit, whereas a low MAPE represents a set curves of similar curves.

The approach aims at defining guidelines for a minimal procedure with limited invasiveness. We focused our analysis on 1) ideal range of saccade amplitude, 2) minimum number of saccades and 3) test-retest reliability.

##### Saccade amplitude

The reliability of the model fitting was evaluated considering subsets of saccades of increasing ranges, within 5, 10, 15, 20, and 25 degrees of eccentricity. For each range, we computed the goodness-of-fit for each model (*R*^2^). The goodness-of-fit was computed on the whole dataset (e.g., saccades at all eccentricities), not just on those used to compute the fit. Exemplifying, each model was fitted over an eccentricity range of 5 degrees, then the obtained *R*^2^ was computed also on the subsets of saccades ranging 10, 15, 20, and 25 degrees. In this way, we evaluated to which extent a model computed on a limited eccentricity range is able to capture and to describe the general performance of the oculomotor system. These values differ from the tested eccentricities (± 1, ± 2, ± 4, ± 8, ± 12, ± 16 or ± 24 deg) to ensure that each group contains enough samples to allow for an effective bootstrap analysis.

##### Minimum number of saccades

To test the reliability of the estimation at increasing number of saccades, we performed a bootstrap analysis considering bootstrap samples of increasing size, from 10 to 100. At each bootstrap, we computed the goodness-of-fit of model (*R*^2^) and the MAPE. We used the Hotelling’s T-squared multivariate test to compare sets of parameters computed for different boot sizes.

##### Test-retest reliability

The reliability of the approach was evaluated by comparing the results obtained from to the first and the second recordings of Exp. 2. For each boot size, we computed the MAPE between each fit from the first recording and all the 1000 fits from the second recording. The resulting 1000×1000 values were then used to compute the median and the first and third quartiles of the MAPE.

#### Oculomotor performance in natural viewing

We wanted to evaluate if a free viewing task, like that in Exp. 2 is suited to estimate the main sequence with the same effectiveness provided by sequential lab testing of Exp. 2. To this aim, we exploited the same analysis used to quantify the test-retest reliability between Exp. 1.1 and Exp. 1.2. In this way, we assessed to which extent a simple, natural, and non-fatiguing task is effective in characterize oculomotor performance.

It is well documented that horizontal saccades are generally faster than vertical and oblique ones, due to a higher performance of the horizontal recti muscles (Vergilino-Perez et al., 2012; Gibaldi et al., 2016). In order to have two sets of data that are actually comparable, we first selected horizontal saccades from Exp. 2 (± 15 deg from the horizontal). The bootstrap analysis was performed also on the whole set of saccades, i.e., directed all around the clock, from Exp. 2. For each bootstrap sample size, we computed the MAPE between each fit from Exp. 2 and all the 1000 fits from Exp. 2. The resulting values where again used to compute the median and the first and third quartiles of the MAPE.

## Results

### Saccade trajectory fitting

This sub-section of the Results analyzes the suitability of the fitting approach to saccadic trajectories together with the influence of sampling frequency.

#### Goodness-of-fit

**Fig. 2:**
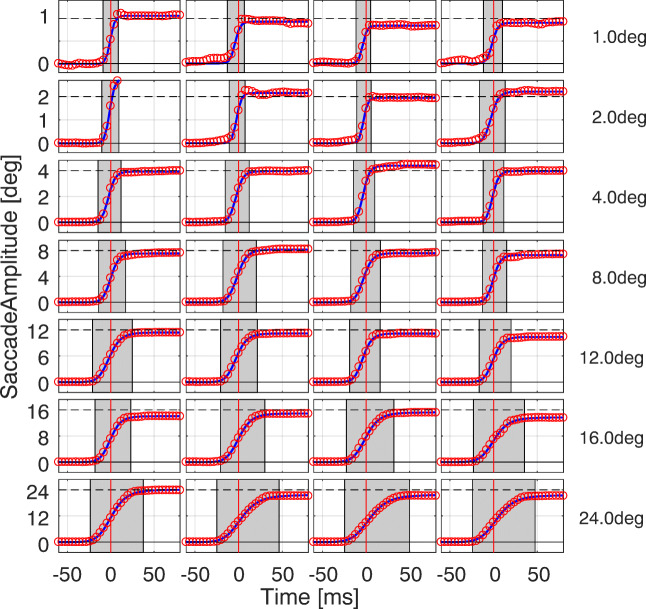
**Model fitting of saccades with different amplitudes.** Each panel reports the absolute trajectory of saccades, randomly chosen from Exp. 2, with the target eccentricity ranging from 1 to 24 degrees. The original data samples (red circles) are shown against the fitted Sigmoid (blue line). The vertical red line locates the instant of peak velocity while the gray patch shows the saccade duration

- Figure 2 shows the effectiveness of the approach for saccades of different amplitudes, randomly chosen from all subjects of Exp. 2. The Sigmoid model is able to represent the trajectory of the saccade regardless its amplitude. We note how saccade duration, highlighted by the gray patch, increases with saccadic amplitude. Since the fit is based on the central part of the saccade only, it is not affected by the presence of possible corrective saccades and overshoots.

Table 2 reports the median and inter-quartile range of *R*^***2***^, computed for each subject for Exp. 2 and 2. The average value of *R*^*2*^ and its limited variability evidence that the proposed model provides an effective description of saccade trajectories. No significant difference in the goodness-of-fit is present between the sequential saccade test (Exp. 2) and the natural exploration (Exp. 2).

**Table 2:** The goodness of fit (R2) for the fitting the saccade trajectory with a Sigmoid function

	Exp. 1.1	Exp. 1.2	Exp. 2
SBJ1	0.9987 ± 0.0020	0.9902 ± 0.0017	0.9971 ± 0.0153
SBJ2	0.9990 ± 0.0119	0.9905 ± 0.0015	0.9963 ± 0.0195
SBJ3	0.9976 ± 0.0023	0.9984 ± 0.0026	0.9974 ± 0.0053
SBJ4	0.9981 ± 0.0032	0.9946 ± 0.0044	0.9881 ± 0.0085
SBJ5	0.9977 ± 0.0053	0.9969 ± 0.0018	0.9979 ± 0.0051
SBJ6	0.9867 ± 0.0041	0.9952 ± 0.0015	0.9898 ± 0.0032
SBJ7	0.9965 ± 0.0058	0.9944 ± 0.0022	0.9949 ± 0.0028
SBJ8	0.9951 ± 0.0048	0.9925 ± 0.0020	0.9937 ± 0.0053
SBJ9	0.9981 ± 0.0039	0.9944 ± 0.0013	0.9952 ± 0.0043

Mean and standard deviation are computed for each subject for the two recordings of Exp. 2 and for 2

#### Sampling Frequency

**Fig. 3:**
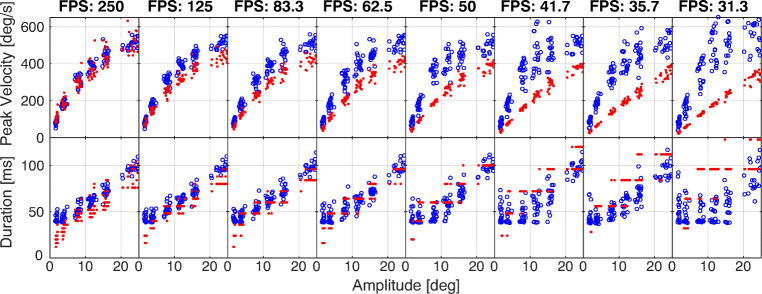
**Effect of sampling frequency on the main sequence.** The panels show the saccadic amplitude (*x-* axis) against peak velocity (*y-* axis, top row) and duration (*y-* axis, bottom row). Red dots are data from the numerical computation method, while blue circles are data computed from analytic solution of the Sigmoid fitting

- Figure 3 shows amplitude, duration, and peak velocity of a set of saccades computed at different sampling frequencies. Red dots refer to the absolute threshold method, while blue circles refer to the Sigmoid fitting. Table 3 reports the p value of the Wilcoxon rank sum test, against the null hypothesis that parameters measured on the original and sub-sampled traces come from a distribution with the same median. Statistically significant values are highlighted in bold characters. The Table also reports the *R*^*2*^ computed between the parameters measured on the original sampling frequency and those measured on the subsampled traces. For the absolute threshold method, we note how the duration suffers from sampling problems, which are even more intense when decreasing the sampling frequency. Likewise, reducing the sampling frequency results in a systematic underestimation of the peak velocity, which is already present at 125 fps. The *R*^*2*^ also shows how duration is the parameter that suffers most from low sampling frequencies.

**Table 3:** Statistical assessment of sampling frequency

			SAMPLING FREQUENCY [Hz]							
			250	125	83.3	62.5	50	41.7	35.7	31.3
AMPLITUDE	num	*p*	1.000	0.966	0.681	0.859	0.977	0.435	0.114	0.067
		***R***^***2***^	1.000	0.998	0.997	0.997	0.995	0.994	0.992	0.989
	fit	p	1.000	0.991	0.987	0.966	0.958	0.959	0.948	0.878
		**R**^***2***^	1.000	1.000	1.000	1.000	1.000	1.000	0.999	0.999
DURATION	num	p	1.000	0.507	0.126	**0.001**	**1e-06**	**1e-10**	**1e-15**	**1e-18**
		**R**^***2***^	1.000	0.847	0.802	0.772	0.761	0.675	0.661	0.462
	fit	p	1.000	0.979	0.957	0.963	0.955	0.173	0.260	**0.004**
		**R**^***2***^	1.000	0.993	0.979	0.956	0.902	0.839	0.887	0.543
PEAK VEL.	num	p	1.000	**0.004**	**0.001**	**1e-06**	**1e-08**	**1e-11**	**1e-14**	**1e-15**
		**R**^***2***^	1.000	0.932	0.924	0.910	0.902	0.906	0.893	0.892
	fit	p	1.000	0.981	0.987	0.951	0.963	0.803	0.557	**0.034**
		**R**^***2***^	1.000	0.997	0.994	0.981	0.930	0.197	0.938	0.864

The Table shows the results of a statistical test to assess the robustness of the measurements of saccadic parameters (amplitude, duration and peak velocity) at decreasing sampling frequency of the eye position data, from 250 Hz (original), down to 31.3 Hz. The used eye movement data are those from Experiment 2. For each parameter, the table reports the p value of a two-sided Wilcoxon rank-sum test, against the null hypothesis that parameters measured on the original and subsampled traces come from a distribution with the same median. Statistically significant values are highlighted in bold characters. The table also reports the coefficient of determination *R*^*2*^, computed between the parameters measured on the original sampling frequency and those measured on the subsampled traces

As expected, the fitting approach is able to provide an estimation of the kinematic parameters of the saccade, which is robust to low sampling frequencies. As a matter of fact, the estimation of amplitude, duration, and peak velocity are reliable down to 50 Hz of sampling frequency.

### Modeling the main sequence

In this sub-section of the Results, we comparatively analyze the nine estimators proposed to model the main sequence (see Table 1).

#### Saccadic Amplitude

**Fig. 4:**
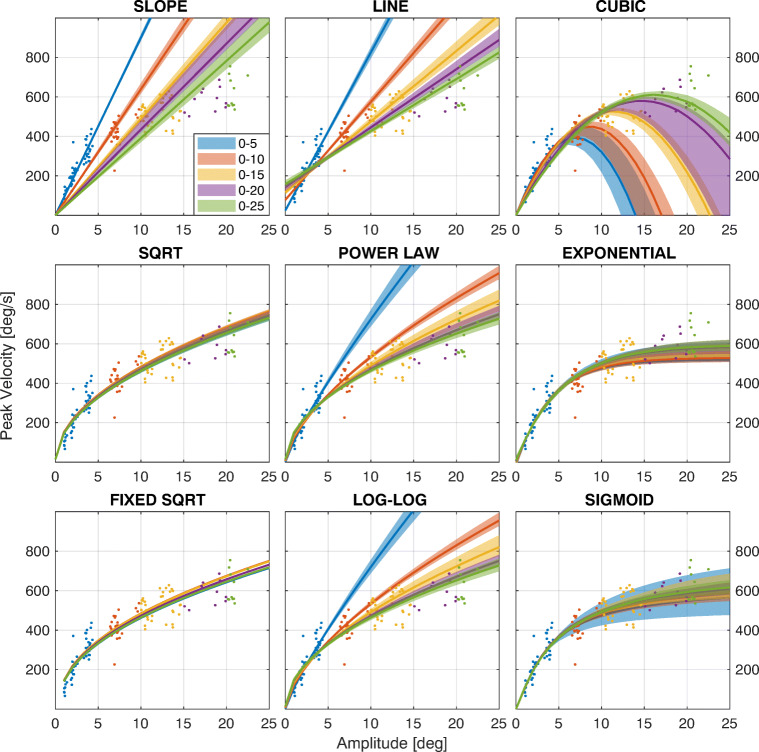
**Model fitting for saccades at different ranges of eccentricity**. Each panel shows the result of the fitting for the nine models considered, using data ranges according to the legend. Data are from SBJ1, Exp 2.1. For each range, the solid line represents the median curve computed over the 1000 bootstraps, while the shaded area represents the 95% confidence interval

**Fig. 5:**
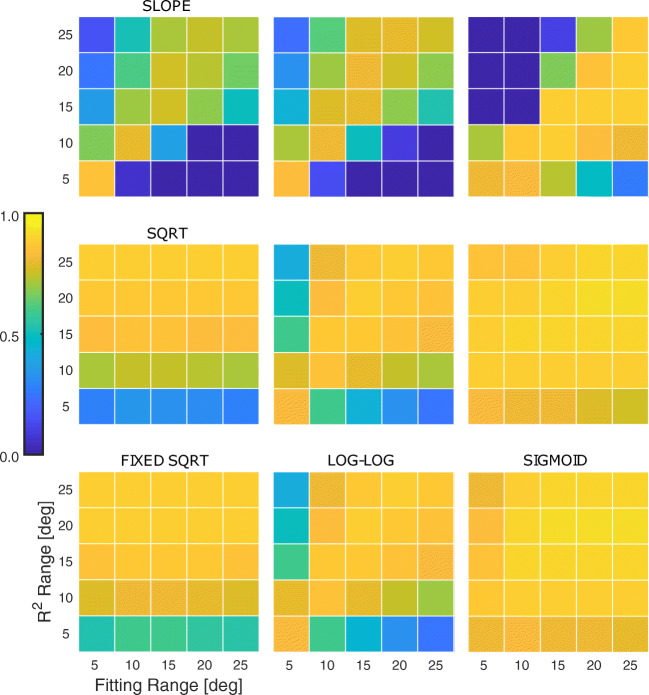
**R**^***2***^ value for model fitting at different saccade ranges. Each panel shows the goodness-of-fit (*R*^*2*^) of the model when considering different ranges of saccades. Data are from SBJ1, Exp. 2.1. The x-axis refers to the maximum range of the subset of saccades used to fit the main sequence. The y-axis refers to the maximum range of the subset of saccades used compute the *R*^*2*^

- Each panel in Fig. 4 shows the result of the fitting for the nine models considered. The fitting has been performed considering different eccentricity ranges for the saccades (see color code in the legend). The solid lines represent the median curves among the 1000 bootstraps, while the shaded areas show the 95% confidence interval. Figure 5 shows the *R*^*2*^ for the fitting. The x-axis represents the saccadic range used to fit the models, while the y-axis is the range used to compute the *R*^*2*^. The results clearly show that for the slope, line and cubic, but also for the power law and log-log models, the estimated model is highly affected by the range considered. This means that these models can be considered reliable at most for the range used to compute the fitting, showing that they have limited generalization capabilities at different saccadic ranges. Conversely, the sqrt and fixed sqrt models provide results that are almost independent of the saccadic range considered. In fact, the result obtained on saccades between 0 and 5 degrees is able to predict the performance of the system at all the considered ranges, also providing a high value of *R*^*2*^ at all ranges. The estimation capability of these two models is generally high and slightly reduced for short saccades (bottom row). The exponential and sigmoid models have an explanatory capability that is almost constant at any range, even if it is slightly lower for saccades smaller than 5 degrees.

#### Minimum number of saccades -

**Fig. 6:**
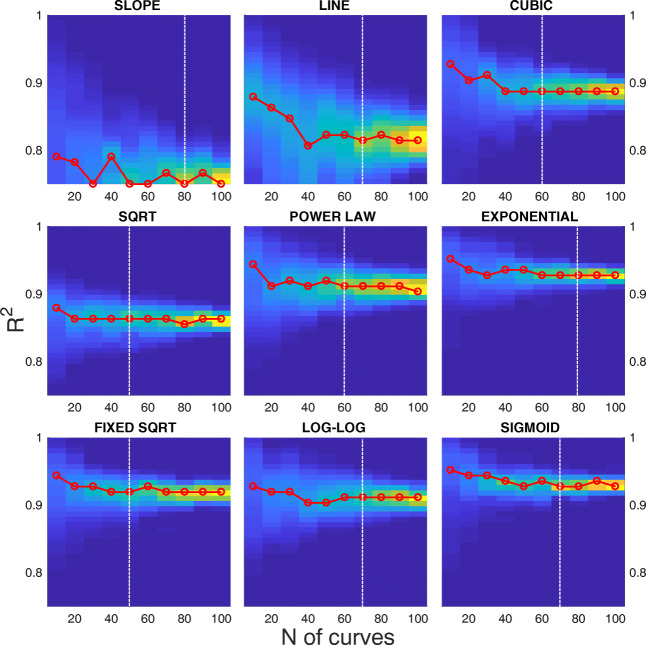
**Goodness-of-fit distribution** (***R***^***2***^) at increasing sample size. Each panel shows the density distribution of the ***R***^***2***^ of the fitting (y-axis) computed over 1000 bootstraps, for different sizes of the boot from 10 to 100 samples (x-axis). Data are from SBJ1, Exp 2.1. The red line represents the maximum of the distribution at each boot size. The white dashed vertical line represents the number of samples beyond which the parameter estimation does not statistically change

**Fig. 7:**
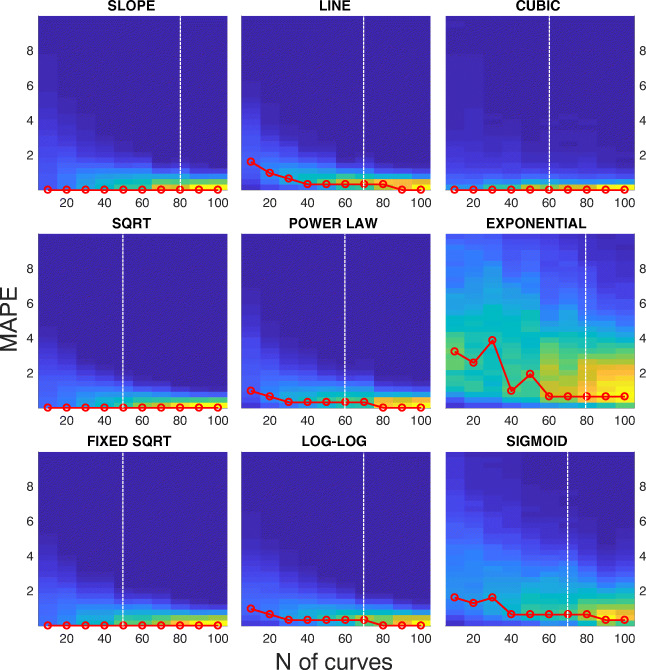
**Distribution of the mean absolute percentage error (MAPE) at increasing sample size.** Each panel shows the density distribution of the MAPE (y-axis). The MAPE is computed considering each possible coupling within the 1000 fitting of the bootstraps, for different sizes of the boot, from 10 to 100 samples (x-axis). Data are from SBJ1, Exp. 2.1. The red line represents the maximum of the distribution at each boot size. The white dashed vertical line represents the number of samples beyond which the parameter estimation does not statistically change

Figure 6 shows the distribution of *R*^*2*^ of the fitting computed over 1000 bootstraps for different sizes of the boot, from 10 to 100 samples. Figure 7 shows the distribution of MAPE on the same set of data. In both figures, the red line highlights the maximum of the distribution at each boot size. The white dashed vertical line represents the boot size beyond which the parameter estimation does not statistically change.

At a first glance, all the models provide high value for *R*^***2***^, above 0.7, and can be considered good estimators of the main sequence. Not surprisingly, the slope and the line models provide poorer performances, since they attempt to provide a linear approximation of a non-linear phenomenon. The models with the highest goodness-of-fit are the exponential and the sigmoid. Again, not surprisingly, models using a higher number of parameters better fit the data. The three-parameter models provide a *R*^***2***^, which is generally higher than 0.9. Interestingly, the fixed sqrt model is able to provide a performance that is comparable to the three-parameter models, even if it relies on a single parameter.

While the choice of one of the three-parameter models seems to be the more reasonable, it is worth considering that a higher number of parameters might produce data over-fitting. Figure 7 provides a complementary perspective of the results. One goal of a good and general estimator is to obtain the same result on a different set of samples from the same distribution, i.e., from the same subject. From this perspective, the MAPE can provide statistics of the robustness of the estimator with a bootstrap analysis. Contrary to *R*^*2*^, the MAPE shows a higher variability of the estimator for a higher parameter number. The 1-parameter models have a variability close to 0 with a narrow distribution even for small sample sizes. The 2-parameter models provide a degraded performance for low sample sizes and the MAPE gradually tends to 0 for increasing sizes. Considering the three-parameter models, the cubic has a performance comparable to the one-parameter models. Besides, the sigmoid and even more the exponential models show higher values of the MAPE and a large variability. This is due to a fitting that varies considerably depending on the data used, and can be interpreted as a tendency to over-fitting. The results from Figs. 6 and 7 are summarized by the white dashed vertical lines, which represent the limit beyond which increasing the number of saccades does not affect the estimation of the main sequence. Exemplifying, for the slope model, increasing the number of saccades in the bootstrap sample from 70 to 80 provides an estimation with no significant difference (p < 0.05). The sqrt and fixed sqrt models, having a limited variability with respect to the other models, can be considered already reliable for a sample size of 50.

### Model sensitivity and test-retest reliability

A mandatory feature for the proposed approach is the sensitivity to a single set of data. Such a feature would allow for comparing different recordings of the same test, like Exp 2.1 and 1.2, but also different test condition like those of Exp. 2 and 2, In this subsection, we will assess the repeatability of the approach, either by repeating the sequential lab testing on different days, or by comparing the results from lab testing with those from natural viewing.

#### Test-Retest in Sequential Saccades -

**Fig. 8:**
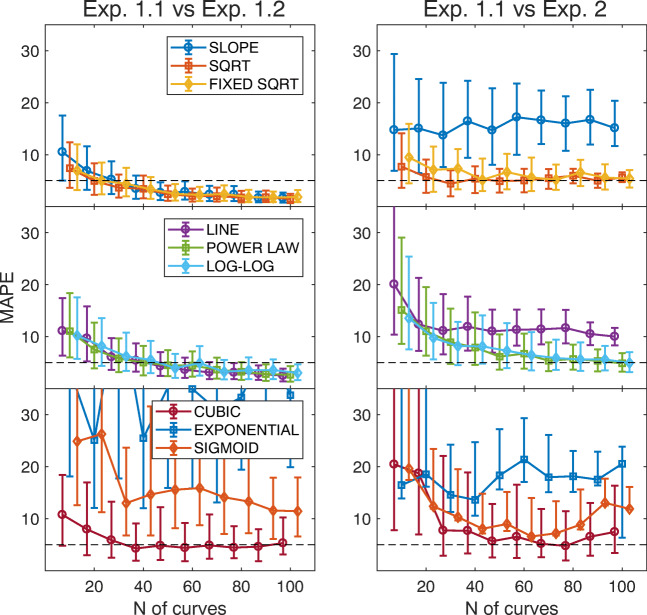
**Test-retest reliability** Each panel shows the minimum absolute percentage error (MAPE) computed when comparing Exp. 1.1 with Exp. 1.2 (left) and when comparing Exp. 1.1 with Exp. 2 (right). Data are from SBJ1. The x-axis shows the number of saccades used to fit the main sequence. The y-axis shows the distribution of the MAPE obtained by the bootstrap analysis (the median and inter-quartile range). Each row shows data for models with a different number of parameters, according to the legends. The horizontal dashed line represents a MAPE equal to 5%, as reference

Figure 8 shows the MAPE computed by comparing data from Exp. 1.1 and Exp. 1.2, for increasing size of the sample bootstrap (left). The mean (solid curves) and 1st and 3rd quartiles (whiskers) have been computed between each fit from the first recording and all the 1000 bootstrap fits from the second recording. From top to bottom, the three panels show data for the one-parameter, two-parameter, and three-parameter models, according to the legends.

This analysis clearly shows how complex models tend to over-fit data. The increase in goodness-of-fit (see Fig. 5) comes at the price of a reduced generalization and repeatability of the measurement. Besides, simpler models are more robust and provide a good repeatability also at small sample numbers, e.g., 50–60 saccades.

#### Free natural gaze exploration vs. sequential saccades

- Figure 10 in Appendix A shows the result of the fitting on the two sets of data from Exp. 2 (blue) and Exp. 2 (orange), for the nine tested models. Figure 8, right, shows the MAPE computed between data from Exp. 1.1, and horizontal saccades from Exp. 2. We observe how the line and slope models provide a different estimate of oculomotor performance, while the cubic model can be considered reliable at small eccentricities.

The power law, log-log, exponential and sigmoid models are able to provide estimates that are numerically similar, but derive from set of parameters that are statistically different between the two sets. Furthermore, the exponential and sigmoid models likely result in data over-fitting, as shown by the considerable variability.

Again, simple models, like sqrt and fixed sqrt, provide the best generalization capabilities. The MAPE is slightly higher but still comparable to that computed between Exp 1.1 and Exp 1.2 (see Fig. 8). Thus, these two models are able to provide repeatability of the estimation across the two methodologies, already for a small boot size.

Table 4 reports the estimation of oculomotor performance on the tested subjects, performed with the fixed sqrt model. As assessed by the test-retest repeatability, the results form Exp. 1.1 and Exp. 1.2 are almost equivalent. The free gaze task of Exp. 2 (selecting horizontal saccades only) provides a slightly lower estimate.

**Table 4:** Oculomotor performance computed from the fixed sqrt model

Subject ID	Exp. 1.1		Exp. 1.2		Exp. 2.0 (hor.)		Exp. 2.0	
	**V**	**R**^**2**^	**V**	**R**^**2**^	**V**	**R**^**2**^	**V**	**R**^***2***^
SBJ1	138.7 ± 1.8	0.857 ± 0.013	138.3 ± 2.1	0.924 ± 0.012	129.7 ± 1.3	0.844 ± 0.004	117.1 ± 4.3	0.923 ± 0.018
SBJ2	138.7 ± 1.7	0.897 ± 0.014	140.2 ± 1.4	0.867 ± 0.005	132.0 ± 1.9	0.925 ± 0.005	121.8 ± 3.7	0.811 ± 0.015
SBJ3	110.4 ± 1.1	0.941 ± 0.004	114.1 ± 1.5	0.922 ± 0.006	121.2 ± 1.9	0.878 ± 0.013	114.5 ± 2.9	0.842 ± 0.011
SBJ4	93.2 ± 0.3	0.919 ± 0.005	87.1 ± 2.1	0.891 ± 0.016	96.1 ± 1.3	0.921 ± 0.014	89.9 ± 1.7	0.913 ± 0.007
SBJ5	100.4 ± 0.6	0.804 ± 0.011	95.6 ± 2.3	0.829 ± 0.020	91.4 ± 0.8	0.940 ± 0.009	81.8 ± 0.9	0.849 ± 0.018
SBJ6	45.4 ± 0.7	0.862 ± 0.013	48.9 ± 0.9	0.812 ± 0.013	47.1 ± 1.2	0.820 ± 0.006	44.3 ± 2.2	0.863 ± 0.009
SBJ7	90.8 ± 2.0	0.923 ± 0.008	88.2 ± 1.8	0.887 ± 0.009	82.5 ± 2.4	0.935 ± 0.011	83.2 ± 2.2	0.852 ± 0.021
SBJ8	77.1 ± 2.3	0.883 ± 0.012	85.1 ± 1.2	0.857 ± 0.013	76.5 ± 0.9	0.941 ± 0.013	72.3 ± 1.8	0.841 ± 0.013
SBJ9	101.7 ± 2.7	0.895 ± 0.007	98.9 ± 3.0	0.897 ± 0.018	103.5 ± 1.6	0.839 ± 0.017	96.2 ± 2.5	0.817 ± 0.011

The Table reports the parameter V of the fixed sqrt model of the main sequence (see Table 1), together with the *R*^*2*^. The median and standard deviation have been computed using bootstrap analysis of 1000 boots, over a sample 100 saccades

#### Model sensitivity -

**Fig. 9:**
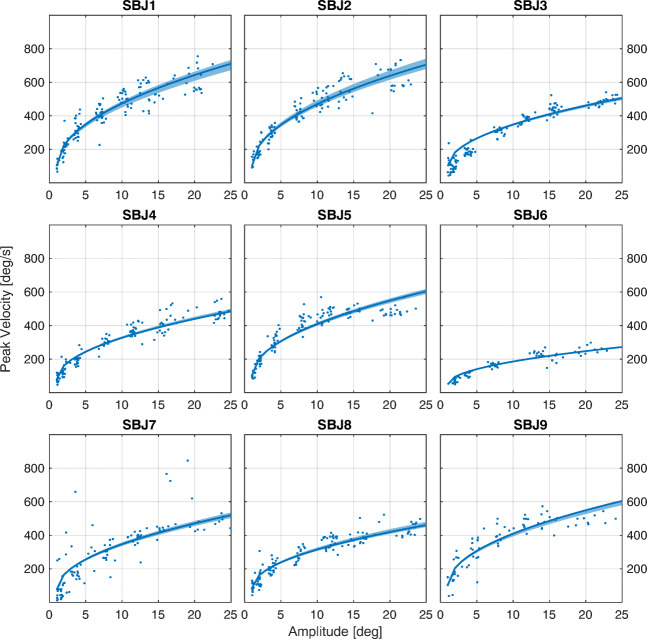
fixed sqrt on sequential saccades task. Each panel shows the result of the fitting for the nine subjects, using the fixed sqrt model. Data are from Exp 2.1. The solid line represents the median curve computed over the 1000 bootstraps for a boot size of 50 samples, while the shaded area represents the 95% confidence interval

As a first qualitative step, the main sequence can be assessed from numerical data about average amplitude, peak velocity, and duration of these saccades, as reported in Table 5. Figure 9 shows the result of the fitting of the fixed sqrt model on data from Exp. 2, for all nine subjects. It is clear how the fitted curves provide an effective description of the data, which is representative of each subject. More interestingly, the fixed sqrt model can effectively describe the data with a single parameter (see Table 4) that captures by itself the oculomotor performance of each subject.

**Table 5:** Statistics of Saccadic Parameters

Subject ID	Amplitude [deg]	Peak Velocity [deg/s]	Duration [ms]
SBJ1	8.80 ± 6.19	408.83 ± 162.35	31.52 ± 14.53
SBJ2	9.08 ± 6.51	413.65 ± 171.42	31.44 ± 15.22
SBJ3	9.86 ± 7.29	300.35 ± 147.23	56.17 ± 24.18
SBJ4	9.58 ± 7.40	290.07 ± 139.37	47.95 ± 22.71
SBJ5	9.37 ± 6.46	370.87 ± 132.20	36.85 ± 22.82
SBJ6	9.03 ± 6.54	160.23 ± 68.23	44.94 ± 20.67
SBJ7	8.91 ± 6.84	295.43 ± 176.46	68.22 ± 25.89
SBJ8	9.42 ± 7.11	281.57 ± 121.97	49.41 ± 27.63
SBJ9	8.96 ± 6.39	358.52 ± 141.46	43.91 ± 27.74

The Table reports the mean and standard deviation for amplitude, peak velocity, and duration for saccades from Exp. 1.1

## Discussion

The main sequence can be employed as a ready-to-use diagnostic tool to assess the integrity of the saccadic system and to eventually provide an explanation of eye movements disorders (Leigh & Kennard, 2004; Ramat et al., 2006). In a seminal work (Bahill et al., 1981), the authors suggested that each lab should create its own normative dataset from healthy subjects. The strong demand for a general approach comes from the recent development and wide-spread use of eye-tracking research, which is demanding standardized and shareable tools for research and clinical applications. Yet, several issues must be considered to get reliable and repeatable measurements. To this aim, we conducted a systematic analysis of the sensitive variables that concur to the collection of robust and reliable measurements, ranging from the sampling frequency of the device, to the choice of the model for the main sequence. With this work, we aim to establish a set of guidelines for a standardized approach to characterize oculomotor performance, which would be robust, general, repeatable, and non-fatiguing.

### Sampling frequency -

Traditionally, a sampling frequency of 330 Hz or even higher is recommended to analyze eye movement traces (Bahill et al.,, 1982, 1983; Juhola et al.,, 1985; Leigh & Zee, 2015) to capture the smallest characteristics of eye movements, and specifically to prevent underestimation of the peak velocity. Though, we claim that such a limit derives from the use of a two-point central difference differentiation algorithm, which is implicitly sensitive to noise and sampling frequency. As a matter of fact, the estimation of saccade kinematics is heavily affected by sampling frequency (see Fig. 3). Assuming that the saccade duration is quite short, a low sampling frequency would provide a sampling period close to the actual duration, thus resulting in a wrong and systematic underestimation of the peak velocity. In a seminal paper (Bahill & Donald, 1983), the authors clearly state that “More complicated algorithms should only be used if their superior performance has been demonstrated”.

The approach we propose is indeed much more complicated than a two-point differentiation. Nevertheless, it comes with a great advantage: this method allows us to heavily relax the 330-Hz requirement, down to 50 Hz. The curve fitting approach mitigates the dependence of the estimation on the sampling frequency. The Sigmoid model is effective in describing the saccadic trajectory with high explanatory capability (see Table 2). Moreover, relying on the mathematical model of the saccade trajectory, the kinematic parameters are obtained with an analytic solution rather than a numerical one. Granted a reasonable accuracy and precision of the device, the proposed methodology allows the use of most of the current commercial low-cost eye-trackers (e.g., see Gibaldi et al., 2017b) for a reliable characterization of oculomotor performance.

### Saccade range -

Our analyses provide clear indications about model selection, depending on the tested range of saccades. If the saccadic range is limited, e.g., less than 5 degrees, the slope model provides a reliable estimation of the main sequence. One must be aware that this model is not capable of generalizing to larger saccadic eccentricities.

On the opposite side, the exponential and sigmoid models provide the highest generalization capability over saccade eccentricity (see Fig. 4). Despite this, the repeatability is relatively poor. Such models could be useful with an extensive dataset from the same subject (i.e., a very large number of saccades), in order to perform a fine characterization of the main sequence.

The model with best generalization and repeatability performances is the sqrt, specifically in the fixed sqrt version. Even if the performance at small ranges is relatively poor, the provided estimation is invariant with respect to the considered range. In fact, the performance measured at short saccades is also able to describe the oculomotor performance at larger saccades, and vice-versa. Beyond offering a high goodness-of-fit, this model also provides low variability within the same bootstrap sample (Fig. 7) and between different measurements (Fig. 8, left). The fixed sqrt is thus able to provide a general and fine characterization of oculomotor performance at normal ranges.

### Number of saccade -

If a model is characterized by a high variability, it also requires a large number of saccades for a stable estimation (e.g., see line and slope, but also sigmoid and exponential).

Provided a reasonable explanatory capability, e.g., a high value of *R*^*2*^, those that require the least number of saccades are the simple models, e.g., sqrt and fixed sqrt. In fact, a limited number of valid saccades (50) is enough to provide a robust characterization of saccadic performance. The same number is also valid for the free viewing task. Since the percentage of horizontal saccades is significantly higher than that for other orientations (e.g., see Gilchrist et al., 2006), a recording time of approximately 2 min would be a safe time to collect enough samples.

### Model Complexity -

Following the general principles of model selection, the simplest models have generally been shown to be the best choice among models at equal performance. A higher number of parameters might provide a higher goodness-of-fit (see Fig. 6), but it comes at the price of a higher complexity and a fit that is more likely to be tailoring the model to the specific dataset. A simple model with similar explanatory ability produces more precise predictions and maintains the capability to generalize. Our choice naturally falls on the fixed sqrt. Aiming at a normative dataset for human eye movements, this model provides a single parameter to characterize the oculo-motor performance, thus allowing for simple and direct comparisons.

### Visual fatigue -

The sequential testing performed in Exp. 2 provides an accurate characterization of eye kinematics, but the task is quite long (10–15 min) and requires sustained attention on the part of the subject. Repetitive re-fixations have been shown to cause fatigue and to slow down saccades (Schmidt et al., 1979; Bahill et al., 1981; Straube et al., 1997; Bollen et al., 1993), and might not be suited for the use with clinical patients. In Exp. 2 we implemented a simple free-viewing task with natural images, which is both non-fatiguing and user friendly. Our results clearly show that the oculomotor performance measured in free viewing is comparable to the estimation obtained on sequential lab experiments (see Fig. 8). For the use with patients, less invasive eye-tracking devices with equivalent precision should be employed, like desktop eye-trackers (e.g., Eyelink 1000 or SMI Red250), or electro-oculographic devices (Lappe-Osthege et al., 2010; Lappe-Osthege et al., 2010).

The free viewing procedure provides a simple and non-fatiguing tool for characterizing the main sequence. Accordingly, it would be well suited for clinical practice to study eye movements on fragile subjects like children or neurological patients. Recent studies on non-collaborative subjects seek to contextually calibrate the device while performing a visual task (Oakes, 2012; Downey et al., 2018). Thus, the proposed experimental procedure could be implemented in parallel to an implicit eye-tracking calibration.

### Sensitivity -

**Fig. 10:**
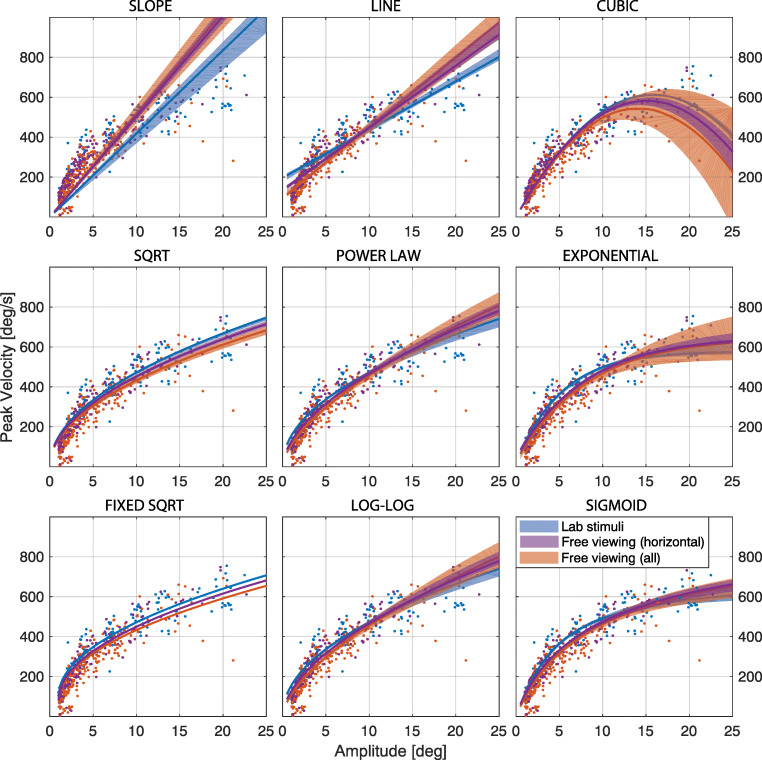
**Test of model fitting on sequential lab testing and free gaze exploration.** Each panel shows the results of the model fitting on data from Exp. 2 (blue), horizontal saccades from Exp. 2 (purple), and saccades in all directions from Exp. 2 (orange), for the nine tested models. Data are from SBJ1. The median curve (solid line) and the 1st and 3rd quartiles have been computed from the bootstrap analysis

Among the tested models, the sqrt and the fixed sqrt provide the highest sensitivity to data. These models are able to provide a compact and accurate representation of each dataset, i.e., of the oculomotor performance of each subject (see Fig. 9), with a high explanatory capability (*R*^*2*^ value always above 0.8, see Table 4). The model reveals a subtle but consistent difference expected between the oculomotor performance of reactive and voluntary saccades: the former is slightly faster than the latter (Gremmler & Lappe, 2017). In fact, the data reported in Table 4 show how our approach is able to capture a higher performance for reflexive saccades, as for Exp. 2, compared to voluntary saccades of Exp. 2. Moreover, horizontal saccades are more rapid than vertical and oblique saccades, due to a higher performance of the horizontal recti muscles (Vergilino-Perez et al., 2012; Gibaldi et al., 2016). The high sensitivity of the proposed approach, and specifically the fixed sqrt model, is able to discriminate such an effect (see Fig. 10), as well as to provide a compact numerical representation of the performance (see Table 4).

### Repeatability -

Few other studies evaluated the repeatability of the main sequence measurement, and generally showed a high variability. For instance, Bollen and colleagues (Bollen et al., 1993) showed a large variability in the estimation between two different sessions. According to our analyses, it is worth considering that their experimental paradigm and their post-processing were not completely suited to the task. First, peak velocity was computed with a two-point central difference algorithm with a sampling frequency of 200 Hz. Such sampling frequency is not sufficient to obtain a reliable measurement of peak velocity (see (Bahill et al., 1981), but also Fig. 3). Second, they used the log-log model over a dataset of 30 saccades. According to our analyses, the chosen model has a considerable variability (Fig. 7), and the authors would have needed a dataset of at least double the size to maximize repeatability. The parameters of saccade kinematics have been shown to have a high repeatability, thus representing an oculomotor signature for a single subject (Bargary et al., 2017). This is particularly true in natural viewing experiments like a pro-saccadic task (Bijvank et al., 2018). Accordingly, a meta-analysis of these parameters, like the main sequence, should have similar reproducibility. In fact, we have shown that the measurement of oculo-motor performance is repeatable, also under different experimental conditions like reactive saccades or voluntary saccades in free viewing. The bootstrap analysis is an ideal method to assess the quality of the measurement in a single dataset. Furthermore, it is useful to assess inter- and intra-subject variability, as well as to compare different experimental paradigms. Besides, it is worth considering that cognitive processing can influence the main sequence, for instance the decision-making under urgency can increase the peak velocity, thus deviating oculomotor performance from the main sequence (Seideman et al., 2018).

### The shape of the main sequence -

Three principal trends can be individuated in the shape of the main sequence, and specifically in the relation between peak velocity and saccadic magnitude: 1) it is roughly linear for small saccades, between 1 degree and 5–10 degrees, 2) it has an inflection point between 10 and 20 degrees, and 3) it smoothly reaches a saturated value for larger saccades. These three characteristics derive from the dynamic characteristics of the eye plant, like friction of the bulb and muscle contraction speed.

We have shown that the fixed sqrt model best captures this behavior, but only under the constraint of saccades larger than 1 degree. Interestingly, this value also corresponds to the accepted value of micro-saccades amplitude (Martinez-Conde et al., 2009). The curve we fit is in fact shifted one degree to the right, and starts from the mean peak velocity for 1-degree saccades, i.e., $\sim 40 deg/s$.

This analysis then leads to the question of what the shape of the main sequence might be for small saccades. Some authors showed that in logarithmic coordinates, the shape is a linear continuum (Martinez-Conde et al., 2009). Our analysis suggests the presence of another inflection (in Cartesian coordinates) between 0.5 and 1 degree of amplitude. It would then be interesting to further investigate the shape of the main sequence, possibly with more accurate devices, like a tracking scanner laser ophthalmoscope, in order to achieve a better accuracy (Sheehy et al., 2012; Bowers et al., 2019).

## Conclusions

In summary, the proposed methodology provides three major contributions to the field of eye movement research.

First, it showed how the measurement of oculo-motor performance is repeatable under different experimental conditions, endorsing the main sequence for a stable characterization of oculomotor performance.

Second, it provides an approach that is relatively insensitive to the sampling frequency of the eye-tracking device, thus allowing the use of some low-cost technologies for an accurate characterization of oculomotor performance.

Third, it provides a thorough assessment of the main sequence models proposed in literature and provides the rationale for a choice.

## Figures and Tables

**Fig. 11:**
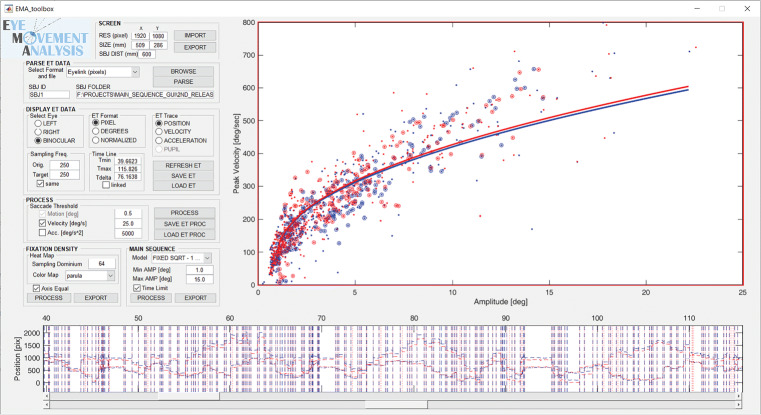
**Computation of the main sequence with the EMA Toolbox.** The figure shows a screenshot of the EMA GUI for the computation of oculomotor performance. The top-right plot shows the main sequence for the left (blue) and the right eye (red). Each dot represents the amplitude (x-axis) and peak velocity (y-axis) of a saccade, while the solid lines represent the fitted main sequence. The data used for the fitting are highlighted by circles

**Fig. 12:**
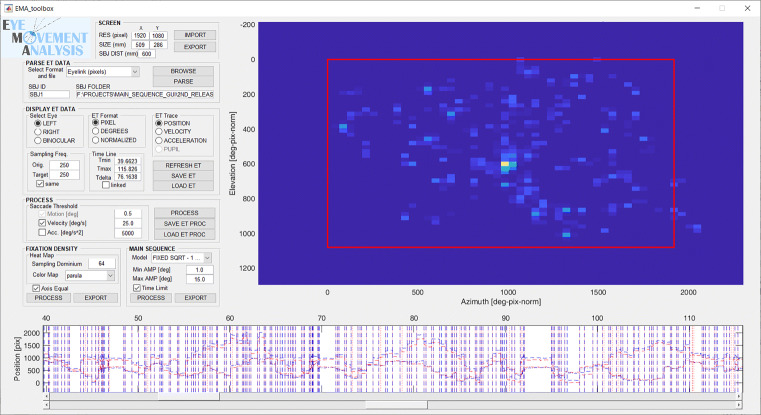
**Computation of fixation density with EMA Toolbox** The figure shows a screenshot of the EMA GUI for the computation of fixation density map. Warm colors represent high density, while cold colors stand for low or no density

## Appendix A: Repeatability over experimental conditions

Figure 10 shows the results from a bootstrap analysis on the nine selected models for the main sequence, comparing Exp. 2 (blue), with horizontal saccades from Exp. 2 (purple), and saccades in all directions from Exp. 2 (orange).

## Appendix B: EMA Toolbox - Eye Movement Analysis

The approach described in this work for the characterization of oculomotor performance has been implemented in a graphic user interface and is released for scientific use. The EMA Toolbox can be downloaded at https://sourceforge.net/projects/ema-toolbox/.

Figures 11 and 12 show the graphic user interface with the possible configurations.

The displayed plots are

- Gaze Data panel - The top panel shows the position, velocity, or acceleration of the eye traces, for the left (blue) and/or the right (red) eye. The unit can be pixel, degrees of visual field or normalized screen coordinates, depending on the configuration. Two sliders below this panel allow to select the desired time window.
- Main Sequence panel - In the bottom right panel shows, each dot represents the amplitude (x-axis) and peak velocity (*y*-axis) of a saccade. The solid lines represent the model fitted to the main sequence, while the circles represent the samples actually used for the fitting.
- Fixation Density panel - The bottom right panel shows the fixation density map computed from the eye position. The red rectangle represents the screen size.

The EMA Toolbox offers a number of possible parameters to modify and customize the processing, at different levels.

- Menu - The button top-left allows to access a menu for the parsing of data files from different eye-tracking devices. For now, the supported formats are *edf/asc* (SR Research Eyelink), *tsv* (SensoMotric Instrument), and *txt*.
- Screen panel
  - The editable fields allow to define the horizontal and vertical resolution (pixel) and size (mm) of the screen.
  - The editable field allows to set the subject distance from the screen itself. At this level, the subject is considered at a fixed and constant distance.
  - The buttons allow to import/export the screen parameters from/to a *ini* file.
- Eye Tracking Data panel
  - Select Eye panel - The radio-buttons allow to show data for the left (blue), right (red) or both eyes.
  - ET format panel - The radio-buttons allow to show data in degrees (default), pixels or normalized to *x* and *y* screen size. and specifically the *x* and *y* position of the eye on screen
  - ET trace panel - The radio-buttons allow to show eye position (*x* and *y*), or velocity, or acceleration (in degrees only).
  - Sampling frequency panel - The editable fields show the original average sampling frequency of the device, and (if desired) a target frequency for resampling the data. The check-box *same* bounds these two values together. The editable fields on the right show and allow to modify the time window selected with the sliders.
  - Refresh/Save/Load ET Data - These three buttons allow to refresh the processed data after some parameter change, save the processed data on disk, and load processed data previously saved.
- Saccade Thresholds panel
  - Motion/Velocity/Acceleration - The editable fields allow to set thresholds on motion, velocity, and acceleration for the identification of saccades.
  - Process ET Data - This button starts the processing of eye-tracking data for fitting the temporal profile on each selected saccade, and extract a number of parameters.
  - Save/Load Saccade Data - These buttons save the saccade data on disk, and load data previously saved.
- Fixation Density panel
  - Sampling dominium - This editable field allows to set the numbers of bins to compute the 2D fixation density histogram. The histogram is computed using a kernel density function (Botev et al., 2010). For computational purposes, this number is rounded to the highest power of 2.
  - Colormap - This pop-up menu allows to choose the color map for the fixation density plot, among the standard Matlab color maps.
  - Axis Equal - This tick-box displays the *x* and *y* axis of the panel proportionally scaled by their actual dimension.
  - Process - This button executes the computation of the fixation density map, and it displays it in the panel to the right. The map is computed using samples between the upper and lower time limits set by the sliders.
  - Export - This button exports the fixation density figure in a standard vector graphic file.
- Main Sequence panel
  - Model - This pop-up menu is for the selection of the model to be fitted to the main sequence. The choice is among the nine models evaluated in this work.
  - Minimum and Maximum Amplitude - These editable fields allow to set an upper and lower limit (in degrees) to select the saccades used to compute the main sequence.
  - Time Limit - If the tick-box is not checked, the main sequence will be computed on all the available saccades identified in the gaze data. Otherwise only those saccades occurring within the time window selected by the sliders will be used.
  - Process - This button executes the computation of the main sequence, and it displays it in the panel to the right.
  - Export - This button exports the main sequence figure in a standard vector graphic file.
